# High expression of DHX9 promotes the growth and metastasis of hepatocellular carcinoma

**DOI:** 10.1002/jcla.24052

**Published:** 2021-10-22

**Authors:** Feng Shi, Shengli Cao, Yaohua Zhu, Qiwen Yu, Wenzhi Guo, Shuijun Zhang

**Affiliations:** ^1^ Department of Hepatobiliary and Pancreatic Surgery The First Affiliated Hospital of Zhengzhou University Zhengzhou China; ^2^ Henan Engineering Technology Research Center for Organ Transplantation Zhengzhou China; ^3^ Zhengzhou Key Laboratory for Hepatobiliary & Pancreatic Diseases and Organ Transplantation Zhengzhou China

**Keywords:** DHX9, epithelial‐mesenchymal transition, hepatocellular carcinoma, metastasis, prognosis

## Abstract

**Background:**

DHX9, an NTP‐dependent RNA helicase, is closely associated with the proliferation and metastasis of some tumor cells and the prognosis of patients, but its role in hepatocellular carcinoma (HCC) is not well‐known. This study was performed to explore the expression and role of DHX9 in HCC.

**Methods:**

The expression of DHX9 in HCC tissues and cell lines was detected by TCGA database, qPCR, western blotting, and immunohistochemistry. The relationship between the DHX9 expression level and the prognosis of patients with HCC was accessed. Then, the function of DHX9 knockdown in HCC cells was examined by CCK‐8, scratch, Transwell, and apoptosis assays. Epithelial‐mesenchymal transition (EMT) was detected by western blotting.

**Results:**

DHX9 was highly expressed in HCC tissues by analyzing both TCGA database and clinical samples. High DHX9 expression level was associated with TNM stage, vascular invasion and metastasis of HCC patients, and was an independent adverse prognostic factor. DHX9 knockdown significantly inhibited cell proliferation, migration, invasion and EMT and increased cell apoptosis in HCC cells.

**Conclusion:**

Our findings suggest that DHX9 participates in the progression of HCC as an oncogene and may be a potential target for the clinical diagnosis and therapy of HCC.

## Graphical abstract

DHX9 expression is upregulated in hepatocellular carcinoma (HCC) tissues. High expression of DHX9 is an independent adverse prognostic factor in HCC. DHX9 promotes the growth and metastasis of HCC cells.

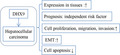

## 1 INTRODUCTION

Liver cancer is one of the most common malignant tumors worldwide, and it ranks sixth in terms of global incidence and third in terms of mortality in 2020, with approximately 906,000 new cases and 830,000 deaths.^1^, ^2^ Hepatocellular carcinoma (HCC) is the most common type of liver cancer (comprising 75%–85% of cases).^1^ In recent years, despite the continuous improvements in the efficacy of surgical treatment and adjuvant chemotherapy, the overall 5‐year survival rate of patients with liver cancer remains between 30% and 40%.^3^ The occurrence of HCC is a complex multi‐step process that involves the imbalance of cell signal transduction pathways, the deletion of DNA repair regulatory gene, the activation of proto‐oncogenes, and the inactivation of tumor inhibitor genes.^4^, ^5^ Exploring new molecular mechanism of the carcinogenesis and development of HCC has an important guiding role in further improving the prognosis of patients with liver cancer.

DHX9, also known as RNA helicase A (RHA), is an NTP‐dependent RNA helicase that belongs to DExD/H‐Box superfamily II of helicases. With an ability to unwind DNA and RNA duplexes, as well as more complex nucleic acid structures, DHX9 appears to play a central role in many cellular processes. Its functions include regulation of DNA replication, post‐transcriptional activation, post‐transcriptional RNA processing, and RNA‐mediated gene silencing.^6^, ^7^ Overexpression of DHX9 is a characteristic of some cancer types, and closely associated with the proliferation and metastasis of tumor cells and the prognosis of patients.^8^, ^9^, ^10^ However, the role of DHX9 in HCC remains unclear.

In this study, the expression of DHX9 in HCC tissues and its function in cells were detected. The correlation between DHX9 level and prognosis of patient with HCC was further analyzed. Our study suggested that DHX9 might be an oncogene and promote cell proliferation, invasion, and metastasis in HCC.

## 2 MATERIALS AND METHODS

### 2.1 TCGA data analysis

Expression profile data from TCGA database were used to analyze the expression characteristics of DHX9 mRNA in 369 cases of HCC and 50 cases of non‐tumor liver tissue, and the correlation between the DHX9 level and prognosis of HCC patient was analyzed by Gene Expression Profiling Interactive Analysis (GEPIA).

### 2.2 Clinical specimens and pathological data

From January 2018 to December 2018, tumor samples and adjacent normal tissues from 69 patients with HCC were collected at the First Affiliated Hospital of Zhengzhou University. In the control group, all normal tissues were derived from paracancerous tissues of the same patient (at least 3 cm from the surgical margin). All samples were confirmed by pathological diagnosis. No radiotherapy, chemotherapy, and hormone therapy were performed before surgery. There were 62 males and 7 females. The patients were aged between 30 and 76 years, with an average age of 58 years. The clinical pathology was classified according to the TNM staging system (AJCC 2010), and included 18 cases of stage I, 17 cases of stage II, 14 cases of stage III, and 20 cases of stage IV disease. The samples were collected with the signed informed consent of all patients, and the research procedure was approved by the Medical Ethics Committee of the First Affiliated Hospital of Zhengzhou University.

### 2.3 RNA isolation and quantitative PCR (qPCR)

Total RNA was extracted from liver tissues using Trizol reagent (Invitrogen) according to the manufacturer’s instructions, and the concentration and the ratio of A260/A280 were determined by Nanodrop. Primers were designed according to gene sequences. *DHX9*: 5’‐CTGTGGCTACAGCGTTCGAT‐3’ (forward primer) and 5’‐GATTCCTCGAATGCCTGCTTC‐3’ (reverse primer). *GAPDH*: 5’‐GTCTCCTCTGACTTCAACAGCG‐3’ (forward primer) and 5’‐ACCACCCTGTTGCTGTAGCCAA‐3’ (reverse primer). SYBR Green Master Mix kit (Promega) and Agilent Mx3005P qPCR System were used for qPCR. Using human GAPDH as an internal reference, the relative expression of genes was calculated using the 2^−ΔΔ^CT method.

### 2.4 Tissue microarray and immunohistochemical analysis

A tissue microarray containing above 69 paired samples was prepared by Wuhan Servicebio Technology Co. Ltd. The chip was gradually dewaxed and hydrated. Citrate buffer was used for antigen retrieval under high temperature and high pressure conditions. After rinsing with PBS, the chip was blocked with goat serum for 10 min at room temperature (RT) and then incubated with primary antibody against DHX9 (Proteintech) overnight at 4°C. After rinsing, the chip was incubated with biotinylated secondary antibody at RT for 1 h, and HRP‐labeled streptavidin and DAB reagent was added after washing with PBS. After washing with tap water, the chip was re‐stained with hematoxylin, dehydrated with gradient ethanol, sealed with gum, and observed under a microscope. The staining intensity was scored by the staining characteristics of the target cells: 0, no staining; 1, yellowish; 2, brownish yellow; and 3, brown. The cell positivity rate was used for scoring: 0, 0%–5%; 1, 6%–25%; 2, 26%–50%; 3, 51%–75%; and 4, >75%. The staining intensity score and the cell positivity rate score were calculated for each tissue on the tissue chip, and the positive comprehensive score was the product of the staining intensity and the positive cell rate.

### 2.5 Western blotting

The tissue and cell proteins were extracted using RIPA lysis buffer. The protein concentration was measured by the BCA method. Equal amounts of protein were loaded and separated on 8%–12% SDS‐PAGE gels and transferred to nitrocellulose membrane, which were then blocked in 5% skim milk at RT for 1 h. The membrane was then incubated with primary antibodies against DHX9, E‐cadherin, Vimentin, matrix metalloproteinase 2 (MMP‐2), MMP‐9, BAX, BCL‐2, Caspase‐3, and GAPDH overnight at 4°C. After rinsing three times with TBST, the membranes were incubated with secondary antibodies diluted at 1:2000 at RT for 1 h. After washing three times with TBST, ECL reagent was added to the membranes for visualization, followed by scanning on BIO‐RAD Imaging System. The protein expression levels of each sample were calculated by Quantity‐One software.

### 2.6 Cell cultures and transfection

The normal liver cell line L02 and seven liver cancer cell lines were purchased from the China Center for Type Culture Collection (Wuhan, China). These cells were cultured in DMEM medium supplemented with 10% fetal bovine serum and were placed in an incubator at 37°C with 5% CO_2_.

DHX9 siRNA and negative control (NC) RNA were transfected to cells using Lipofectamine 3000 reagent according to the instructions of siRNA purchased from Gene Pharma Co., Ltd. The effect of transfection was verified by western blotting.

### 2.7 Cell Counting Kit‐8 (CCK‐8) assay

Exponential phase cells were diluted and seeded into 96‐well plates at a density of 2.0 × 10^3^ cells/well and cultured for 4 days. At 0, 24, 48, 72, and 96 h, cell proliferation was detected by CCK‐8 assay (TIANGEN Biotech Co., Ltd.) and the absorbance at 450 nm was detected by spectrophotometer after adding CCK‐8 reagent for 2 h.

### 2.8 Transwell assay

The invasion and metastasis of cells were detected by Transwell assay. To detect cell migration, HCC cells in the exponential phase were inoculated in the upper chambers of Transwell plates (Chemicon, USA) at a density of 3 × 10^4^ in 200 μl medium. Then 700 μl normal medium was added to the lower chambers and cultured for 24 h. To detect cell invasion, diluted Matrigel (BD, USA) was placed in the upper chambers of the Transwell plates and incubated at 37°C for 1 h. After hydration of the basement membrane with DMEM, the cells were evenly inoculated in the upper chambers of the Transwell plates at a density of 3 × 10^4^ in 200 μl, and 700 μl normal medium was added to the lower chambers. After 24 h of routine culture, the cells in the lower chambers were fixed and stained with 0.5% methanol crystal purple solution for 30 min. After washing with PBS, the chamber was placed on the slide, and five visual fields were randomly selected under a microscope for imaging.

### 2.9 Scratch assay

To detect the migration ability of HCC cells, HCC cells were inoculated in six‐well plates at a density of 1 × 10^6^ per well. RNA interference was performed when the cells had adhered to the plates and had reached a confluency of approximately 60%. After successful transfection, a vertical scratch was made using pipette tips, and was observed after 0, 24, and 48 h. Five different migration points on both sides of the scratch were selected, and the average distance was measured.

### 2.10 Flow cytometric analysis of cell apoptosis

Cell apoptosis was analyzed with an Annexin V apoptosis detection kit (Yeasen Biotech Co., Ltd.). Approximately 1 × 10^6^ cells were collected and rinsed with PBS. The collected cells were resuspended in 500 μl binding buffer. Later, 5 μl Annexin V‐Alexa Fluor 647 and 10 μl propidium iodide (PI) were introduced into 100 μl of the cell suspension, and additional cultivation was performed at room temperature for 15 min without light. Finally, the stained cells were added 400 μl PBS, and the late and early apoptotic rates were determined using flow cytometry (Agilent, USA).

### 2.11 Statistical analysis

The statistical analysis was performed by SPSS 22.0 software. The Student’s t test was used to compare the difference between the two groups. The Kaplan‐Meier method and log‐rank test were used for survival analysis. The correlation between clinical factors and prognosis was analyzed by univariate and multivariate analysis. All data were expressed as mean ± SD. *p *< 0.05 was considered statistically significant.

## 3 RESULTS

### 3.1 The expression of DHX9 is increased and correlated with prognosis of HCC patients based on TCGA database

**FIGURE 1:**
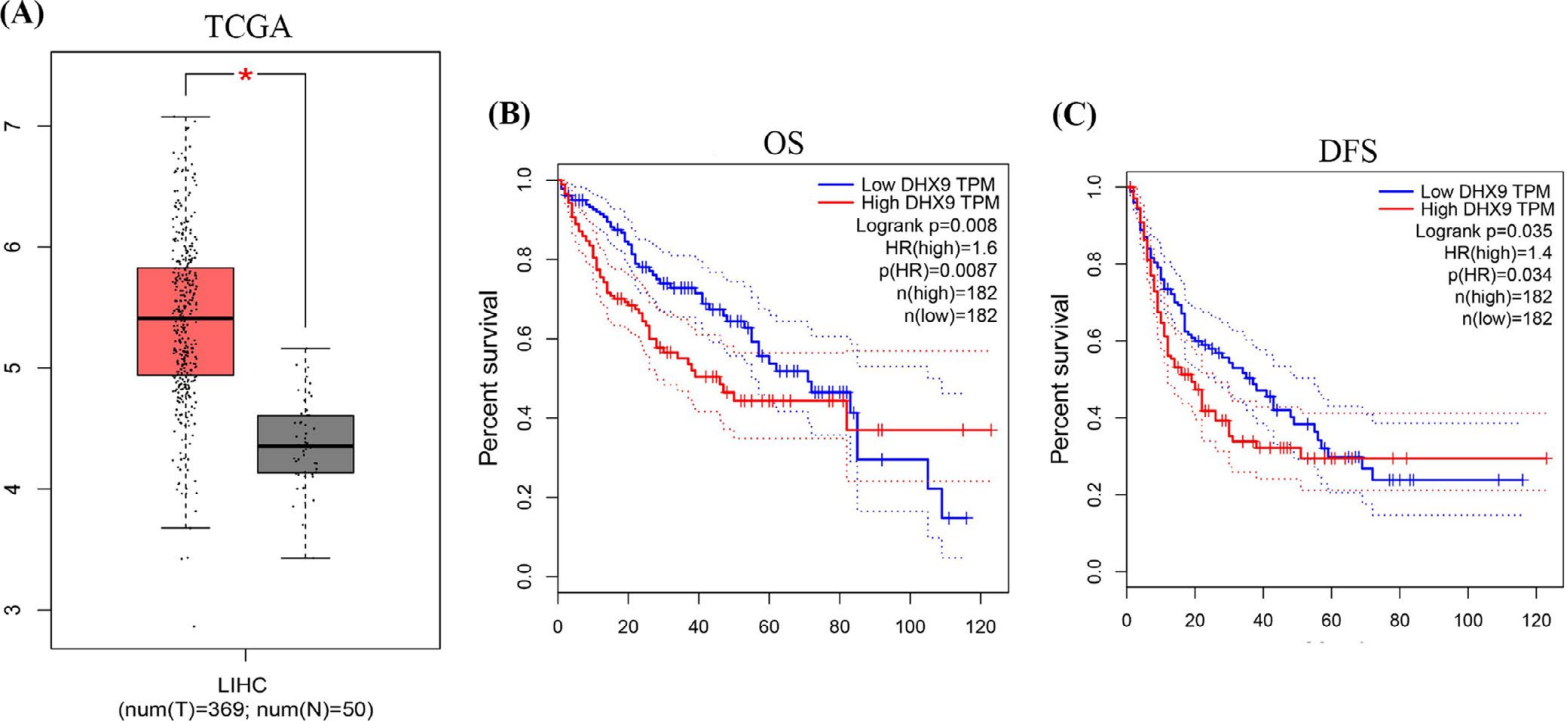
DHX9 expression is associated with prognosis of HCC patients in TCGA. (A) In the TCGA database, DHX9 expression was increased in 369 HCC tissues compared with that in 50 normal liver tissues. T, tumor tissues, N, normal tissues. *p < 0.05. (B–C), High level of DHX9 in HCC predicted adverse OS (B) and DFS (C) compared with group with low level, which was evaluated using Kaplan‐Meier survival analysis in 182 paired HCC tissues

We firstly analyzed the expression of DHX9 in 369 cases of HCC and 50 cases of non‐tumor liver tissues based on TCGA database, and found that the expression of DHX9 in HCC was significantly higher than that in non‐tumor tissues (Figure 1A). In addition, survival analysis showed that HCC patients with high DHX9 expression levels showed more adverse overall survival (OS) and disease‐free survival (DFS) than patients with low DHX9 expression level (Figure 1B,C).

### 3.2 DHX9 mRNA and protein are highly expressed in HCC tissues

**FIGURE 2:**
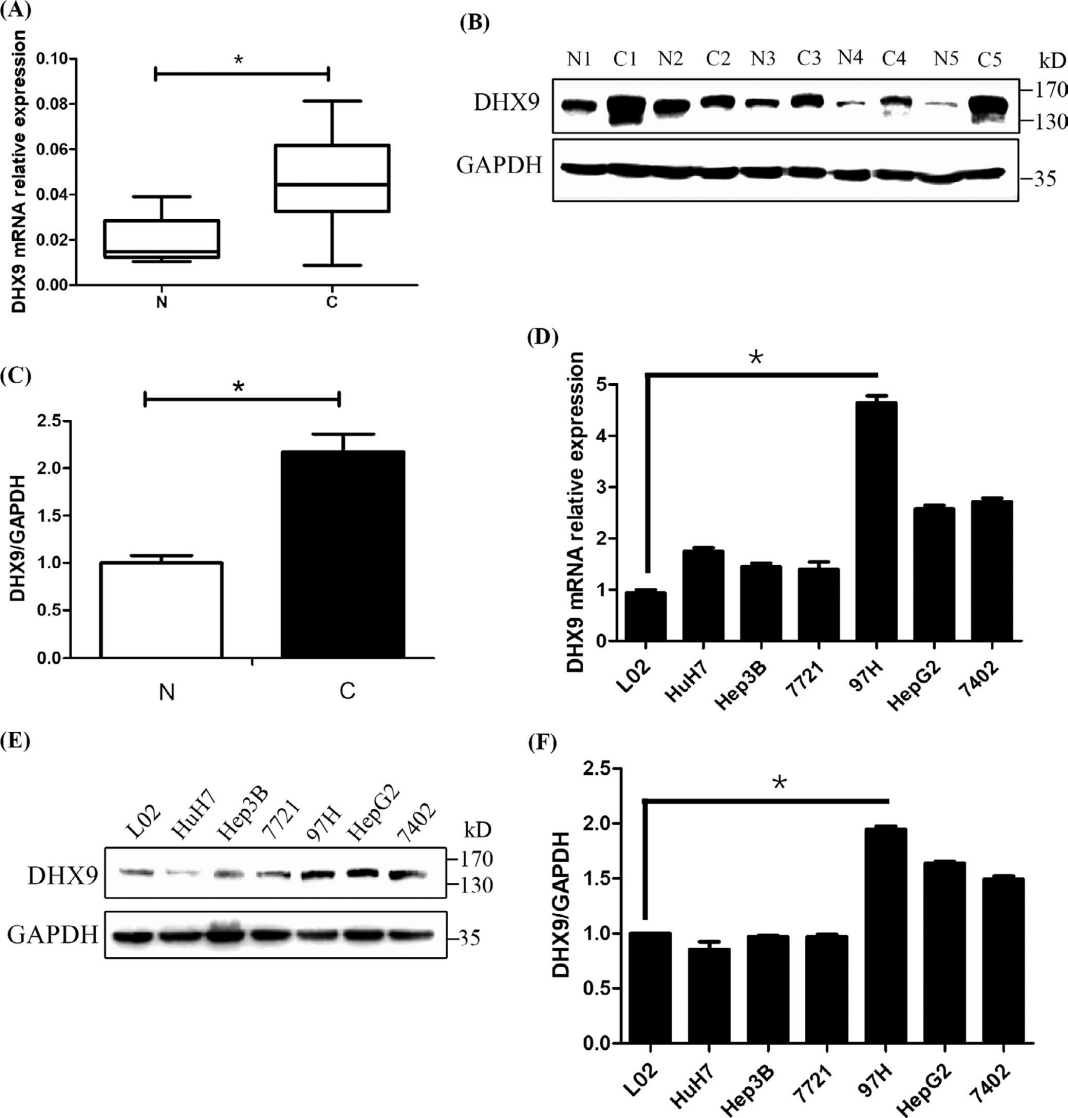
DHX9 expression is upregulated in HCC tissues. (A) The mRNA expression of DHX9 was detected by qPCR in 69 paired HCC and their paracancerous tissues. The expression levels of DHX9 were significantly upregulated in HCC tissues. (B) The protein levels of DHX9 in tissues were analyzed with western blotting. (C) Quantification of western blotting results in 69 paired HCC and their paracancerous tissues, Protein levels were normalized against GAPDH. (D) Relative mRNA expressions of DHX9 in six HCC cell lines and normal hepatocytes L02 were analyzed with qPCR. (E) The protein levels of DHX9 in six HCC cell lines and normal hepatocytes were detected with western blotting. (F) Quantification of western blotting results in normal hepatocytes and six HCC cell lines. Data are expressed as mean values ± SD, *p < 0.05. N, normal tissues, C, carcinoma tissues

We collected 69 cases of clinical samples from our hospital, and the expressions of DHX9 in HCC and paracancerous tissues were detected by qPCR and western blotting. As shown in Figure 2A–C, the mRNA and protein expression of DHX9 in HCC tissues was significantly higher than that in paired paracancerous tissues.

The levels of DHX9 were further detected in a normal immortalized liver cell line L02 and HCC cell lines. It was found that DHX9 mRNA and protein in MHCC97H, HepG2, and BEL‐7402 were significantly upregulated compared with L02 cells, but unchanged in Huh‐7, Hep3B, SMMC‐7721 (Figure 2D–F). The expression of DHX9 was highest in the highly invasive HCC cell line MHCC97H, and therefore MHCC97H was selected for follow‐up knockdown experiments.

### 3.3 The expression of DHX9 is correlated with clinicopathological parameters of HCC patients

**FIGURE 3:**
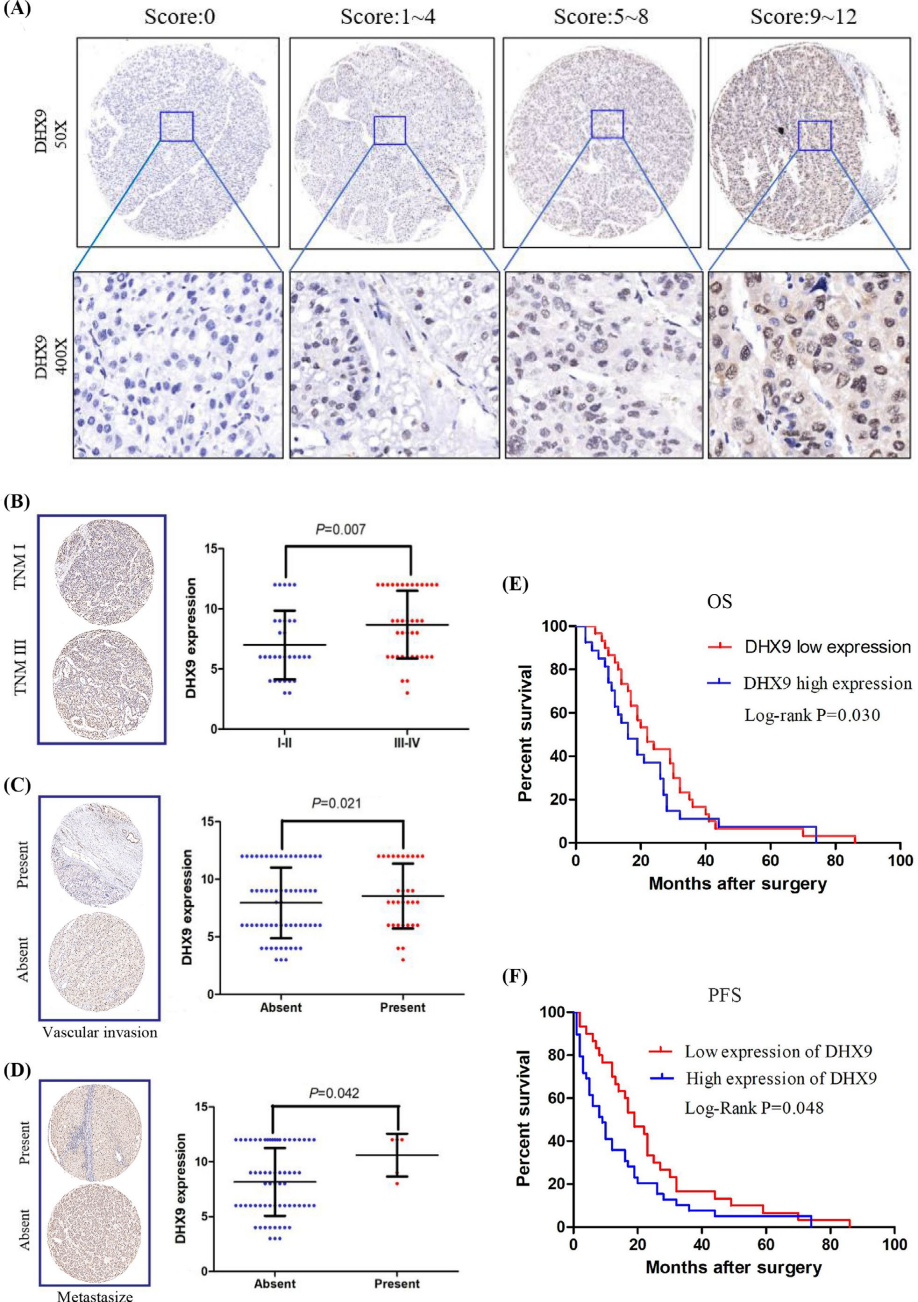
DHX9 expression is correlated with clinicopathologic features of HCC patient. (A) Representative pictures of immunohistochemistry staining of DHX9 in HCC tissues were indicated. (B–D) The relationship between DHX9 expression and TNM stage (B), vascular invasion (C), and metastasis (D) of HCC patients was indicated respectively. (E–F) Kaplan‐Meier analysis of OS (E) and PFS (F) of HCC patients according to DHX9 expression level

To further define the correlation between the expression level of DHX9 and clinicopathological parameters in HCC tissues, a microarray containing 69 paired tissues was prepared and analyzed. The results of immunohistochemistry showed that DHX9 protein was mainly expressed in the cytoplasm of hepatocytes, and its expression in HCC tissues was significantly higher than that in paired paracancerous tissues (Figure 3A). Based on the immunohistochemistry results, DHX9 expression was associated with TNM stage, vascular invasion, and metastasis (Table 1 and Figure 3B–D). HCC patients with high DHX9 expression level showed more adverse OS and progression‐free survival (PFS) than patients with low DHX9 expression level (Figure 3E,F), which was consistent with the result of TCGA data analysis.

**TABLE 1:** The relationship between DHX9 expression and the clinicopathologic features of HCC patients

Clinical features	*n*	DHX9 expression		χ^2^	*p* value
		Low	High		
Gender					
Male	62	26	36	0.592	0.442
Female	7	4	3		
Age (yeas)					
<50	39	18	21	0.261	0.609
≥50	30	12	18		
Tumor size (cm)					
<5	16	7	9	0.001	0.980
≥5	53	23	30		
AFP (μg/L)					
<400	34	15	19	0.011	0.916
≥400	35	15	20		
TNM stage					
I&II	31	19	12	7.267	0.007**
III&IV	38	11	27		
Tumor differentiation					
Poor	10	6	4	1.299	0.254
Moderate/Well	59	24	35		
Cirrhosis					
Absent	13	6	7	0.047	0.829
Present	56	24	32		
LN metastasis					
Absent	67	30	37	1.584	0.208
Present	2	0	2		
Vascular invasion					
Absent	50	26	24	5.366	0.021*
Present	19	4	15		
Recurrence					
Absent	36	18	18	1.303	0.254
Present	33	12	21		
Metastasis					
Absent	64	30	34	4.147	0.042*
Present	5	0	5		

* *p* < 0.05.

** *p* < 0.01.

Prognosis of the HCC patients was obviously correlated with tumor size (*p *= 0.011), differentiation degree (*p *= 0.047), lymph node metastasis (*p *= 0.004), vascular invasion (*p *= 0.0001), metastasis (*p *= 0.003), and DHX9 expression level (*p *= 0.032) in univariate analysis. Additionally, multivariate analysis exhibited that DHX9 high level (*p *= 0.045), tumor size (*p *= 0.043), and vascular invasion (*p *= 0.038) were the independent adverse prognostic factors (Table 2).

**TABLE 2:** Univariate and multivariate analysis of prognostic factors in HCC patients for OS

Clinical features	*n*	Univariate analysis		Multivariate analysis	
		*p*	HR(95%CI)	*p*	HR(95%CI)
Gender					
Male	62	0.900	1.052 (0.476–2.323)		
Female	7				
Age (yeas)					
<50	39	0.163	1.428 (0.866–2.355)		
≥50	30				
Tumor size (cm)					
<5	16	0.011*	2.254 (1.209–4.200)	0.043*	1.991 (1.021–3.885)
≥5	53				
AFP (μg/L)					
<400	34	0.148	1.445 (0.878–2.378)		
≥400	35				
TNM stage					
I&II	31	0.109	1.507 (0.9132.488)		
III&IV	38				
Tumor differentiation					
Poor	10	0.047*	1.466 (1.005–2.140)	0.791	1.060 (0.687–1.636)
Moderate	39				
Well	20				
Cirrhosis					
Absent	13	0.870	0.949 (0.505–1.784)		
Present	56				
LN metastasis					
Absent	67	0.004**	9.57 (2.078–44.153)		
Present	2				
Vascular invasion					
Absent	50	0.001**	2.560 (1.450–4.519)	0.038*	1.974 (1.037–3.755)
Present	19				
Recurrence					
Absent	36	0.664	1.116 (0.681–1.829)		
Present	33				
Metastasis					
Absent	64	0.003**	4.30 (1.621–11.451)	0.309	1.815 (0.576–5.717)
Present	5				
DHX9 expression					
Low	30 39	0.032*	1.734 (1.049–2.865)	0.045*	1.668 (0.988–2.814)
High					

* *p* < 0.05.

** *p* < 0.01.

### 3.4 DHX9 knockdown inhibits HCC cell proliferation, migration, and invasion

**FIGURE 4:**
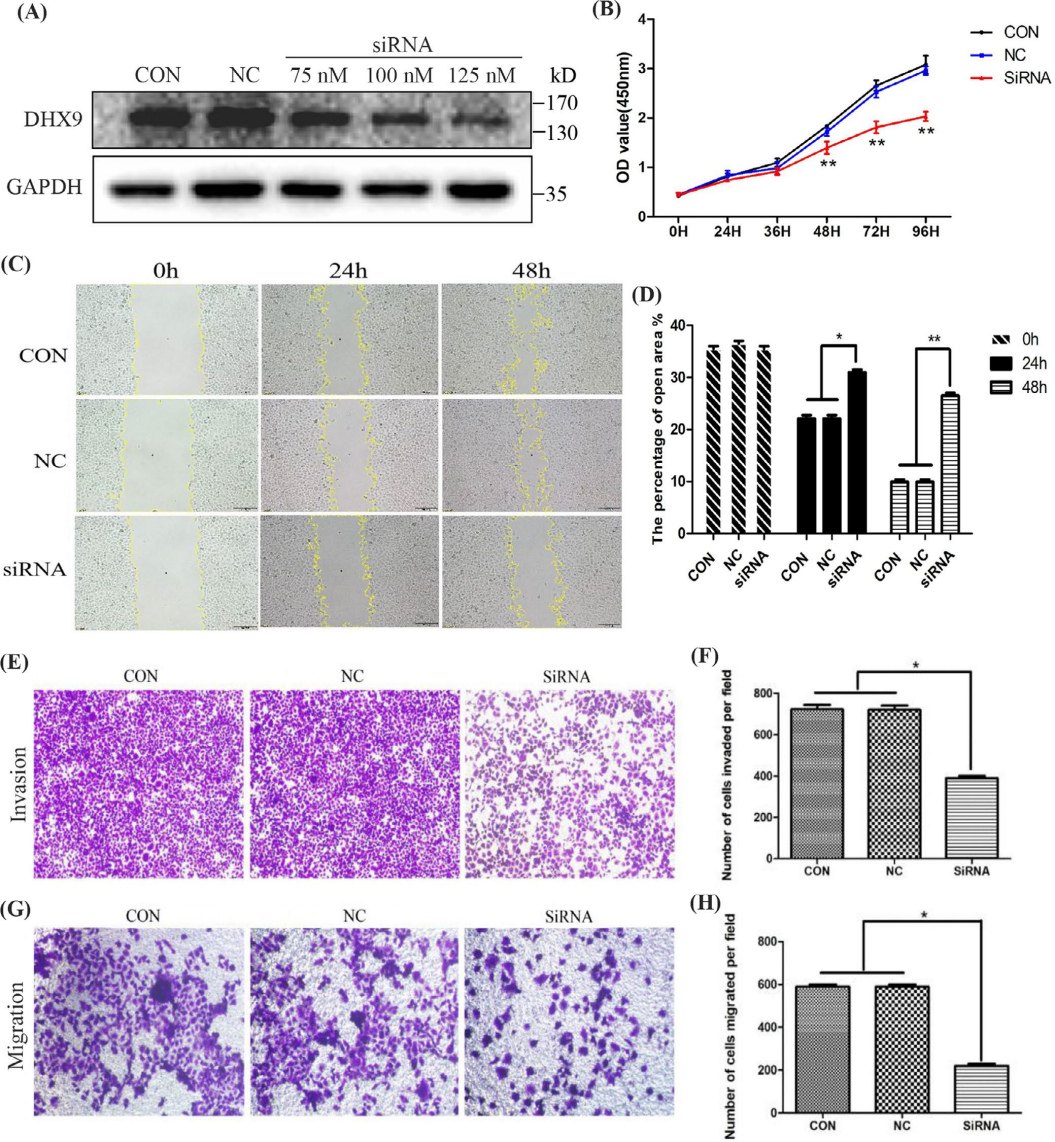
DHX9 knockdown inhibits HCC cell proliferation, migration and invasion. (A) The silencing effect of DHX9 siRNA on cells was analyzed with western blotting. The silencing was obvious when 125 nM siRNA was transfected. (B) Cell proliferation was determined by CCK‐8 assay. DHX9 siRNA could inhibit proliferation obviously at 48, 72, and 96 h after treatment. (C) Cell migration monitored by scratch assay at 0, 24, and 48 h. (D) Wound areas were calculated using Image‐Pro Plus software, and the areas of DHX9 siRNA group were increased at 24 and 48 h. (E) Transwell migration assays on the indicated cells. (F) The number of migrated cells was quantified by counting five independent fields. (G) Transwell invasion assays on the indicated cells. (H) The number of invasive cells was quantified. The numbers of migrated and invasive cells were increased in the DHX9 siRNA group. *p < 0.05. CON, control group without any infection. NC, infected with negative lentivirus; siRNA, infected with DHX9‐siRNA. *p < 0.05

To verify the function of DHX9 in HCC cells, we selected MHCC97H cells with high DHX9 expression for siRNA knockdown assay. Western blotting showed that the silencing effect of siRNA was significant when 125 nM siRNA was transfected (Figure 4A). CCK‐8 assay showed that DHX9 knockdown significantly inhibited the proliferation of MHCC97H cells at 48, 72, and 96 h (Figure 4B). In the scratch assay, DHX9 siRNA‐treated cells covered almost 73% of scratches in 24 h and at least 85% in 48 h, while the cells in the NC group covered only 18% in 24 h and 25% in 48 h (Figure 4C,D). Transwell assays showed that the migration rate of cells in the DHX9 siRNA group was slower than that in the control and NC groups, and the number of invaded cells of the DHX9 siRNA group was significantly increased (Figure 4E–H).

### 3.5 DHX9 knockdown promotes apoptosis and inhibits epithelial‐mesenchymal transition in HCC cells

**FIGURE 5:**
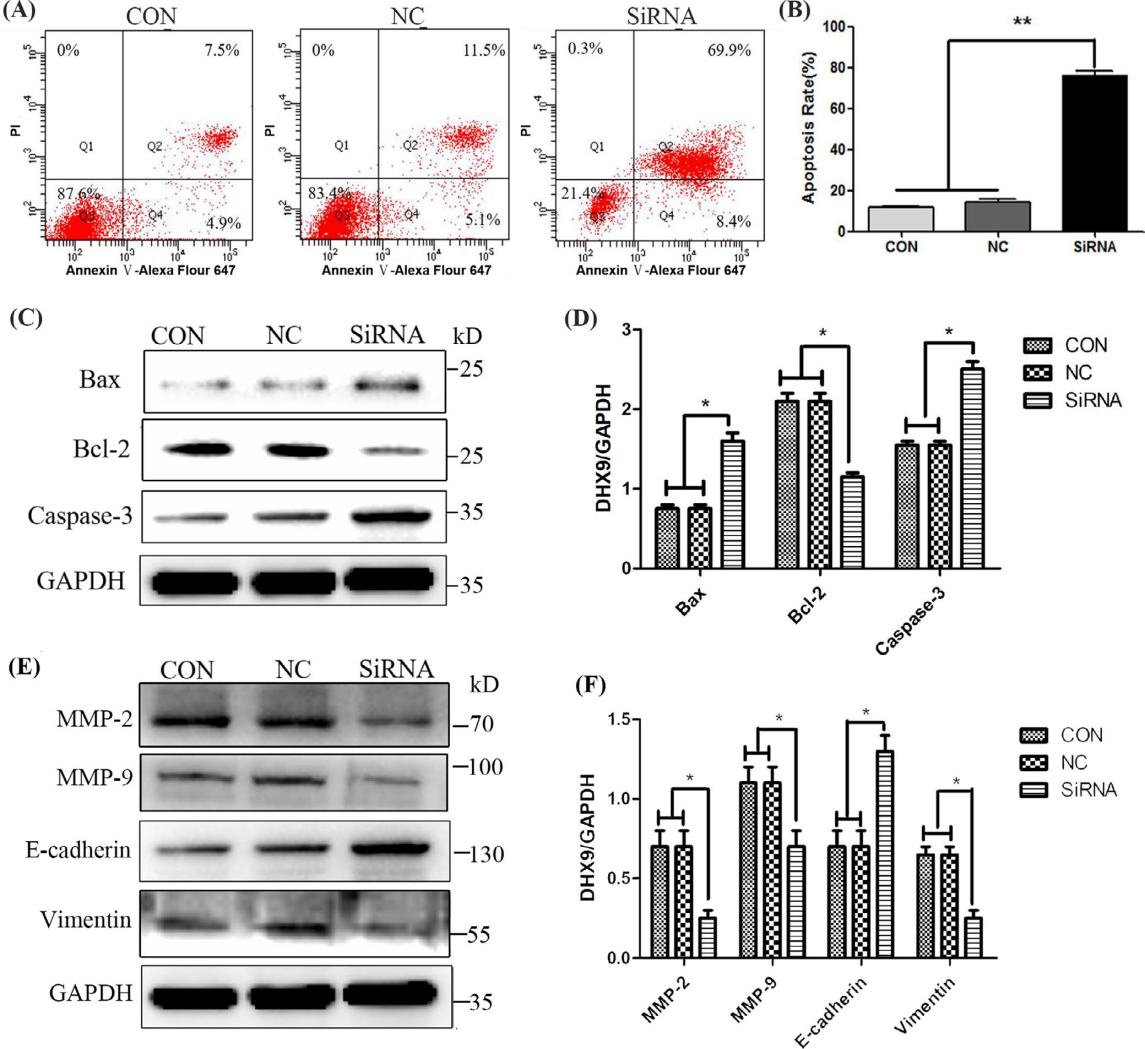
DHX9 knockdown promotes cell apoptosis and inhibits EMT in HCC cells. (A) Apoptosis cells were stained with AnnexinV‐Alexa Fluor 647/PI and analyzed using flow cytometry. (B) Quantitative analysis of flow cytometry showed that the apoptosis cells in the DHX9 siRNA group were significantly increased. (C) Apoptosis‐related proteins were detected with western blotting. GAPDH was used as a control. (D) Quantitative results of western blotting showed that DHX9 siRNA enhanced the levels of Bax and Caspase‐3 and inhibited the level of Bcl‐2. (E) The protein levels of E‐cadherin, Vimentin, MMP‐2, and MMP‐9 were detected by western blotting. GAPDH was used as a control. (F) Quantitative results of western blotting showed that DHX9 siRNA enhanced the expression of E‐cadherin and decreased the expressions of Vimentin, MMP‐2, and MMP‐9 compared with the control and NC group.*p < 0.05.

The effect of DHX9 knockdown on the apoptosis of HCC cells was assessed by flow cytometry with AnnexinV/PI staining. The results showed that knockdown of DHX9 could significantly promote the apoptosis of MHCC97H cells (Figure 5A,B). Simultaneously, western blotting showed knockdown of DHX9 could significantly increase the expression of apoptosis‐promoting protein BAX and Caspase‐3 and decrease the expression of apoptosis‐inhibiting protein BCL‐2 (Figure 5C,D).

Epithelial‐mesenchymal transition (EMT) is one of the important mechanisms of the invasion and metastasis of tumor cells. We detected EMT markers by western blotting and found that knockdown of DHX9 could promote the expression of the epithelial marker E‐cadherin and decrease the expression of mesenchymal markers Vimentin, MMP‐2 and MMP‐9 (Figure 5E,F).

## 4 DISCUSSION

Because of the central role of DHX9 in gene regulation and RNA metabolism, there are growing implications for DHX9 in human diseases.^6^ Much effort has been expended lately in characterizing the association between DHX9 dysregulation and cancer development; however, there is still conflict as to whether it regularly functions as an oncogene or tumor suppressor.^11^ The effect of DHX9 on cancers appears to be cell‐type specific, dependent on the kind of binding partners.^11^, ^12^ Some studies suggested that DHX9 functioned as an oncogene and highly expressed in cancer tissues.^13^ Palombo et al.^14^ showed that poison‐exon inclusion in DHX9 by hnRNPM and SRSF3 reduced its expression and inhibited cell proliferation in Ewing sarcoma malignancy. Can et al.^15^ found that DHX9 was overexpressed in the serum and tissues of lung cancer, and promoted the progression of lung cancer. Cheng et al.^16^ demonstrated that minichromosome maintenance protein 2 (MCM2)/MCM3‐DHX9 axis had an important role in osteosarcoma progression, and DHX9 promoted osteosarcoma cell proliferation. On the contrary, others suggested that DHX9 acted as a tumor suppressor. Yan et al.^8^ demonstrated that DHX9 inhibited cell proliferation, migration, invasion, and EMT in lung adenocarcinoma cells by regulating STAT3. Chenet al.^17^ indicated that DHX9 acted as a downstream mediator of KIF1Bβ to play tumor‐suppressor function in neuroblastoma. Halaby et al.^18^ suggested that DHX9 acted as a p53 IRES trans‐acting factor to increase expression and synthesis of p53 and inhibited breast cancer development.

The role of DHX9 in regulating the occurrence and development of HCC remains to be unclear. Wang et al.^19^ identified an oncogenic lncRNA in HCC, named lnc‐UCID, and disclosed that lnc‐UCID enhanced CDK6 expression by competitively binding to DHX9 and sequestering DHX9 from CDK6‐3'UTR. Yu et al.^20^ discovered a tumor suppressor circRNA‐cSMARCA5 that was downregulated transcriptionally by DHX9 in hepatoma cells. Though it has been reported largely that DHX9 can function as a partner to regulate the expression of some gene or non‐coding RNA in cancer, the expression and key role of DHX9 itself in HCC remain to be studied further.

In this study, we firstly analyzed TCGA database and found that the expression of DHX9 was upregulated in HCC tissues and associated with the prognosis of the patients. Then, using clinical samples and cell lines collected at our center, we verified that DHX9 mRNA and protein were highly expressed in HCC tissues and some cell lines. In addition, the results of tissue microarray also confirmed that DHX9 expression was associated with TNM stage, vascular invasion, and metastasis. DHX9 high level was an independent adverse prognostic factor. In the cellular functional assay, we confirmed that DHX9 knockdown could significantly inhibit cell proliferation, migration, invasion and EMT, and promote apoptosis in HCC cells. These results reveal that DHX9 is highly expressed in HCC and functions as an oncogene to promote tumor cell growth and impact the prognosis of HCC patients. However, we only performed functional assay in MHCC97H cells due to its highest DHX9 expression level. Some cell lines didn’t display increased DHX9 expression which was possibly related to the heterogeneity of cells. In addition, we also explored the related mechanism of DHX9 in HCC, and detected the activity of JAK/STAT3, PI3K/AKT, MAPK/ERK, and NF‐κB signaling, but no definite and meaningful results were obtained (data not shown). In the next study, more molecular mechanisms of DHX9 need to be explored and verified in vitro and in vivo.

In summary, our results demonstrate that DHX9 is highly expressed in HCC tissues, correlates with the survival and prognosis of patients, and promotes the proliferation, invasion, and metastasis of HCC cells. Therefore, DHX9 has an important role in promoting the occurrence and development of HCC and may be a potential therapeutic target and diagnostic biomarker of HCC.

## CONFLICT OF INTEREST

All authors declare that they have no conflict of interests.

## Data Availability

The data that support the findings of this study are available from the corresponding author upon reasonable request.

## References

[1] Sung H , Ferlay J , Siegel RL , et al. Global cancer statistics 2020: GLOBOCAN estimates of incidence and mortality worldwide for 36 cancers in 185 countries. CA Cancer J Clin. 2021;71(3):209‐249. 33538338 10.3322/caac.21660

[2] Siegel RL , Miller KD , Fuchs HE , Jemal A . Cancer statistics, 2021. CA Cancer J Clin. 2021;71(1):7‐33. 33433946 10.3322/caac.21654

[3] Hartke J , Johnson M , Ghabril M . The diagnosis and treatment of hepatocellular carcinoma. Semin Diagn Pathol. 2017;34(2):153‐159. 28108047 10.1053/j.semdp.2016.12.011

[4] Yang C , Huang X , Liu Z , Qin W , Wang C . Metabolism‐associated molecular classification of hepatocellular carcinoma. Mol Oncol. 2020;14(4):896‐913. 31955511 10.1002/1878-0261.12639 PMC7138397

[5] Zhang B , Wu H . Decreased expression of COLEC10 predicts poor overall survival in patients with hepatocellular carcinoma. Cancer Manag Res. 2018;10:2369‐2375. 30122986 10.2147/CMAR.S161210 PMC6078074

[6] Chakraborty P , Huang J , Hiom K . DHX9 helicase promotes R‐loop formation in cells with impaired RNA splicing. Nat Commun. 2018;9(1):4346. 30341290 10.1038/s41467-018-06677-1 PMC6195550

[7] Leone S , Bar D , Slabber CF , Dalcher D , Santoro R . The RNA helicase DHX9 establishes nucleolar heterochromatin, and this activity is required for embryonic stem cell differentiation. EMBO Rep. 2017;18(7):1248‐1262. 28588071 10.15252/embr.201744330 PMC5494521

[8] Yan X , Chang J , Sun R , et al. DHX9 inhibits epithelial‐mesenchymal transition in human lung adenocarcinoma cells by regulating STAT3. Am J Transl Res. 2019;11(8):4881‐4894. 31497206 PMC6731401

[9] Hou P , Meng S , Li M , et al. LINC00460/DHX9/IGF2BP2 complex promotes colorectal cancer proliferation and metastasis by mediating HMGA1 mRNA stability depending on m6A modification. J Exp Clin Cancer Res. 2021;40(1):52. 33526059 10.1186/s13046-021-01857-2 PMC7851923

[10] Yuan D , Chen Y , Yang Z , et al. SPOP attenuates migration and invasion of choriocarcinoma cells by promoting DHX9 degradation. Am J Cancer Res. 2020;10(8):2428‐2445. 32905556 PMC7471363

[11] Gulliver C , Hoffmann R , Baillie GS . The enigmatic helicase DHX9 and its association with the hallmarks of cancer. Future Sci OA. 2020;7(2):FSO650. 33437516 10.2144/fsoa-2020-0140 PMC7787180

[12] Lee T , Pelletier J . The biology of DHX9 and its potential as a therapeutic target. Oncotarget. 2016;7(27):42716‐42739. 27034008 10.18632/oncotarget.8446 PMC5173168

[13] Ding X , Jia X , Wang C , et al. A DHX9‐lncRNA‐MDM2 interaction regulates cell invasion and angiogenesis of cervical cancer. Cell Death Differ. 2019;26(9):1750‐1765. 30518908 10.1038/s41418-018-0242-0 PMC6748089

[14] Palombo R , Verdile V , Paronetto MP . Poison‐exon inclusion in DHX9 reduces its expression and sensitizes ewing sarcoma cells to chemotherapeutic treatment. Cells. 2020;9(2):328. 10.3390/cells9020328 PMC7072589 32023846

[15] Cao S , Sun R , Wang W , et al. RNA helicase DHX9 may be a therapeutic target in lung cancer and inhibited by enoxacin. Am J Transl Res. 2017;9(2):674‐682. 28337295 PMC5340702

[16] Cheng DD , Zhang HZ , Yuan JQ , et al. Minichromosome maintenance protein 2 and 3 promote osteosarcoma progression via DHX9 and predict poor patient prognosis. Oncotarget. 2017;8(16):26380‐26393. 28460433 10.18632/oncotarget.15474 PMC5432265

[17] Chen ZX , Wallis K , Fell SM , et al. RNA helicase A is a downstream mediator of KIF1Bbeta tumor‐suppressor function in neuroblastoma. Cancer Discov. 2014;4(4):434‐451. 24469107 10.1158/2159-8290.CD-13-0362

[18] Halaby MJ , Harris BR , Miskimins WK , Cleary MP , Yang DQ . Deregulation of internal ribosome entry site‐mediated p53 translation in cancer cells with defective p53 response to DNA damage. Mol Cell Biol. 2015;35(23):4006‐4017. 26391949 10.1128/MCB.00365-15 PMC4628062

[19] Wang YL , Liu JY , Yang JE , et al. Lnc‐UCID promotes G1/S transition and hepatoma growth by preventIng DHX9‐mediated CDK6 down‐regulation. Hepatology. 2019;70(1):259‐275. 30865310 10.1002/hep.30613 PMC6618099

[20] Yu J , Xu QG , Wang ZG , et al. Circular RNA cSMARCA5 inhibits growth and metastasis in hepatocellular carcinoma. J Hepatol. 2018;68(6):1214‐1227. 29378234 10.1016/j.jhep.2018.01.012

